# Comment: Comments on ‘Flow-injection analysis— an idea incomplete?’

**DOI:** 10.1155/S1463924683000036

**Published:** 1983

**Authors:** Harris I. Tarlin

**Affiliations:** Control Equipment Corporation 171 Lincoln Street Lowell Massachusetts 01851 USA


unsegmented liquids along tubes for a very long time. However, it has required the perception of workers such as Růžička and Hansen [6] and Stewart *et al.* [7] for the realization that this apparently simple operation was capable of a remarkable degree of exploitation. It was, in fact, the prolific work of Růžička and Hansen [1 and 2] that led us to investigate the potential of FIA in clinical chemistry. Using simple systems constructed from items of equipment commonly used in clinical analyses, we have been able to carry out analyses with economy, speed and accuracy and with negligible carry-over. For those familiar with continuous-flow analysis, the speed of FIA is startling: with fast reactions the analysis may be complete 10 s after introduction of the sample.

Holy suggests that there is a need in FIA for turbulent flow in which the detector may recognize the sample as a square wave. Apart from the difficulty of achieving turbulent flow, it is problematical whether or not flow injection would work effectively under such conditions. Mixing of the sample slug with the carrier stream of reagent depends on the fact that it moves under conditions of laminar flow. The slug adopts a hollow bullet shape and travels along a boundary layer which forms a constantly replenished source of reagent. With the narrow-bore tubing in use, radial diffusion of reagent into slug is extremely rapid. These conditions would not be obtained with turbulent flow.

Holy's commentary [5] contains a number of other misleading statements regarding the practice of FIA. Contrary to his assertions, we find from our own experience and by recourse to the literature that FIA calibration curves are usually linear, that triplicate (or duplicate) measurements are unnecessary and that separation steps (when necessary) can be carried out at least as fast as when using segmented-flow analysis (SFA).

If a single-channel FIA system is compared with a comparable SFA system for carrying out an assay in which the sample reacts rapidly with reagent, it will be evident that:

(*a*) The FIA system will be ready for use almost immediately, whilst the SFA system requires several minutes of operation before its base-line is established.

(*b*) The FIA peaks will be available first—usually within 30 s of sample injection, as against some minutes for SFA.

(*c*) The FIA system will consume less reagent and normally less sample (even after allowing for the small volume which is needed to wash the valve through). The technique known as merging zones [8], in which slugs of both sample and reagent are borne by carrier streams of distilled water and merged downstream of the pump, provides the ultimate in economy. No segmented-flow system can begin to match this.

Clearly, with these fast reactions the FIA approach is much superior; fortunately a number of the most commonly requested tests in clinical chemistry involve such fast reactions.

For many other tests in the clinical field, kinetic assay techniques are preferred. A unique feature of the non-segmented approach is that the flow can be stopped and the sample zone arrested in the cuvette whilst the reaction progresses. The rate of reaction can thus be measured. This cannot be achieved with SFA because the elasticity of the compressed gas in the bubbles will cause movement to continue after the pump is stopped.

In the case of end-point analyses in which relatively long incubations are required (for example, several minutes), SFA systems are still preferable since they offer a faster rate of analysis than can be achieved with FIA. However, in clinical chemistry laboratories such analyses are nowadays relatively rare.

In practice, the weakest link in FIA has been the need to use valves to introduce the sample into the carrier stream. These require some waste of sample and in our hands have been prone to develop leaks. Recently we have developed a number of fully automatic valve-less machines in which there is no waste of sample [9]. These machines can be left on standby indefinitely, they make use of merging zones and they can, when desired, be operated in the stopped-flow kinetic mode.

We have successfully run some 20 different clinical chemistry analyses at 150 samples/h on these machines.

Clinical chemists are beginning to demand selective multichannel analysers which, in contrast to the traditional profile machines, carry out only those tests specifically requested. Between demands these new machines remain on standby. Selective machines so far available have all been based on discrete analysis, but an FIA system such as the one we describe would lend itself perfectly to operation in the selective mode. Because it cannot be operated intermittently there is no place for SFA in this type of machine.

Holy argues that the vast number of SFA machines sold over the last 25 years proves the excellence of the system. The Model T Ford sold in vast numbers in its day, but this is not an argument to condemn the modern motor-car. SFA had the field to itself when it was introduced; the manufacturers of FIA machines, on the other hand, face well-entrenched opposition from established machines and a steadily deepening recession.

### References

1. Růžička, J. and Hansen, E., *Analytica Chimica Acta*, **114** (1980), 19.

2. Růžička, J. and Hansen, E., *Flow Injection Analysis* (Wiley-Interscience, New York, 1981).

3. Ranger, C., *Analytical Chemistry*, **53** (1981), 20a.

4. Rocks, B. and Riley, C., *Clinical Chemistry*, **28** (1982), 409.

5. Holy, H. W., *Journal of Automatic Chemistry*, **4** (1982), 111.

6. Růžička, J. and Hansen, E., *Analytica Chimica Acta*, **78** (1975), 145.

7. Stewart, K. K., Beecher, G. R. and Hare, P. E., *Analytical Biochemistry*, **70** (1976), 167.

8. Bergamin, H. F°., Zagatto, E. A. G., Krug, F. J. and Reis, B. F., *Analytica Chimica Acta*, **101** (1978), 17.

9. Riley, C., Aslett, L. H., Rocks, B. F., Sherwood, R. A., Watson, J. D. McK. and Morgon, J., *Clinical Chemistry* (in press).

**C. Riley and B. F. Rocks**

*School of Engineering and Applied Sciences, University of Sussex and Biochemistry Department, Royal Sussex County Hospital, Brighton BN2 5BE, UK*

### Comments on 'Flow-injection analysis—an idea incomplete?'

This commentary addresses the many inaccuracies and innuendoes which appeared in a recent article by H. W. Holy [1]. In his article, Holy states that flow-injection analysis (FIA) has very limited acceptance and impact 'since commercial instruments have been available since 1959'. The fact is that mass-produced FIA instruments and *significant* marketing efforts for these instruments began around 1978. Thus, it is a bit early to be discussing 'impact' and 'acceptance'. Holy further states that the goal of FIA is 'achievable—theoretically'. The rather large number of FIA journal articles, plus the availability of a comprehensive text containing more than 100 references, negates the suggestion that FIA exists—'theoretically' only.

Mr Holy comments that FIA solvent extractions are carried out at 'low analysis rates', while calibration curves are 'rarely linear'. I recently carried out the analysis of Vitamin $B_1$ using the FIA solvent-extraction method described by Karlberg [2] at a sampling rate in excess of 100 samples/h. I have not yet observed in the literature any significant number of non-linear FIA calibration curves, nor have I observed *any* non-linear calibration curves in the FIA methods that I have developed.

The concept of having to use more samples with FIA than with other systems is totally *incorrect*. After being involved with segmented-flow analysers for more than 14 years, and FIA for most of its commercial history, I have never had the need to run more samples or standards with FIA. In fact, quite to the contrary! Because of the high reliability and precision of the FIA method, the need for repetitive analysis is minimal and certainly no greater than with other commercial instruments.

Mr Holy alleges that 'the simplicity has been lost with FIA' and questions whether FIA offers advantages over commercial methods. I have had occasion to convert several segmented-flow analysis methods to FIA. Putting aside the savings in terms of rapid method development due to low 'dead times', rapid sample throughput and small reagent consumption, a typical converted procedure offers the following advantages over current commercial methods:

(1) All injected air-lines are eliminated. (And thus the classical 'bubble pattern' problem is eliminated.)
(2) In several instances, the dialyser has been eliminated. (And thus with it the elimination of all its inherent problems such as leakage.)
(3) Elimination of several mixing coils and, in some cases, some reagent lines which were proven to be unnecessary.

The resulting flow diagram offers a degree of economy and simplicity which has yet to be found in segmented-flow analysis.

Throughout his article, Mr Holy misses the point by questioning whether FIA offers any unique advantages. He poses the question: 'Is there really any advantage in analysing metals by FIA rather than by atomic absorption?' A better question would have been, 'Is there an advantage to using FIA with AA or ICP?' The many unique advantages of using FIA with flame photometric instruments have been discussed by Greenfield [3]. I have personally observed the use of FIA/AA and FIA/ICP on samples with high salt content which yielded quantitative results but could not be tested in the absence of FIA due to high noise levels.

I hope the above comments will assist analysts in evaluating one of the most important separations: viz. the separation of fact from fiction.

### References

1. Holy, H. W., *Journal of Automatic Chemistry*, **4** (1982), 111.

2. Karlberg, B. and Thelander, S., *Analytica Chimica Acta*, **114** (1980), 129.

3. Greenfield, S., *Industrial Research and Development* (1981), 140.

**Harris I. Tarlin**
*Control Equipment Corporation, 171 Lincoln Street, Lowell, Massachusetts 01851, USA*

**Copyright Clearance Center**
21 Congress Street
Salem, Massachusetts 01970
(617) 744-3350
a not-for-profit corporation

Participation in the Copyright Clearance Center (CCC) assures you of legal photocopying at the moment of need. You can:

*Fill requests for multiple copies, interlibrary loan (beyond the CONTU guidelines), and reserve desk without fear of copyright infringement.*

*Supply copies simply and easily from registered publications.* The CCC's flexible reporting system accepts photocopying reports and returns an itemized invoice. *You need not keep any records,* our computer will do it for you.

The Copyright Clearance Center is your one-stop place for on-the-spot clearance to photocopy for internal use. You will never have to decline a photocopy request or wonder about compliance with the law for any publication registered with the CCC.

Circle No. 3 on Reader Enquiry Card

NEW FROM **SHIMADZU**

**GC-8A Series GAS CHROMATOGRAPHS**

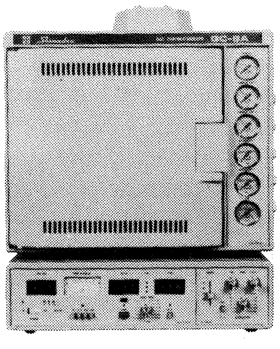

***Dual FID** With quartz jets and cylindrical collectors

***Dual TCD** Constant current type with differential (semi-diffusion) flow and preamplifier. Full filament protection.

***Column Oven** Programmable or Isothermal. Low thermal mass for quick analytical cycling. Glass, metal or capillary columns. Two stage overheat protection.

*Independent heating of Injection Ports/Detectors.

*Digital temperature setting.

*On column injection.

*Full flow controls (mass flow controllers are standard on carrier gases).

*Plus **SHIMADZU's** world renowned reputation for exceptional reliability and high performance.

*At a price you can afford.

**We are always available to advise on the most suitable equipment for any application and to demonstrate in a user's laboratory. We particularly welcome visitors to our headquarters where we can demonstrate the practical application of our comprehensive range of scientific instrumentation.**

Sunderland House, Station Road, Hetton, Houghton le Spring, Tyne & Wear DH5 0AT England
Telephone: Hetton (0783) 260452 & 260433 Telex: 53689
**DYSON INSTRUMENTS**

Circle No. 4 on Reader Enquiry Card

